# Management of Cerebellar Tonsillar Herniation following Lumbar Puncture in Idiopathic Intracranial Hypertension

**DOI:** 10.1155/2015/895035

**Published:** 2015-01-18

**Authors:** Kenneth R. Hoffman, Sean W. Chan, Andrew R. Hughes, Stephen J. Halcrow

**Affiliations:** ^1^Intensive Care Unit, Canberra Hospital, Yamba Drive, Canberra, ACT 2605, Australia; ^2^Neurology Department, Canberra Hospital, Yamba Drive, Canberra, ACT 2605, Australia; ^3^Neurosurgical Unit, Canberra Hospital, Yamba Drive, Canberra, ACT 2605, Australia

## Abstract

Lumbar puncture is performed routinely for diagnostic and therapeutic purposes in idiopathic intracranial hypertension, despite lumbar puncture being classically contraindicated in the setting of raised intracranial pressure. We report the case of a 30-year-old female with known idiopathic intracranial hypertension who had cerebellar tonsillar herniation following therapeutic lumbar puncture. Management followed guidelines regarding treatment of traumatic intracranial hypertension, including rescue decompressive craniectomy. We hypothesize that the changes in brain compliance that are thought to occur in the setting of idiopathic intracranial hypertension are protective against further neuronal injury due to axonal stretch following decompressive craniectomy.

## 1. Introduction

Idiopathic intracranial hypertension (IIH) causes symptoms and signs of raised intracranial pressure (ICP) without focal neurological signs. It has an overall incidence of 1.6/100,000 per annum [1], an unclear pathogenesis, although it has significantly higher incidence in obese females aged 20–44 at 19/100,000 [2].

Lumbar puncture (LP) is classically contraindicated in the setting of raised ICP due to the risk of cerebellar tonsillar herniation. However, LP is routinely performed both for diagnostic and therapeutic purposes in IIH [3]. It has been postulated that the reason herniation does not occur in this setting is a reduction in brain compliance due to persistently high ICP [4].

To date two case reports of herniation have been reported in IIH following LP, although both of these patients had focal neurological signs on presentation and a fatal outcome [5].

We report a case of herniation following LP in a patient with IIH, her subsequent intensive care management, and clinical course.

## 2. Case Report

**Figure 1:**
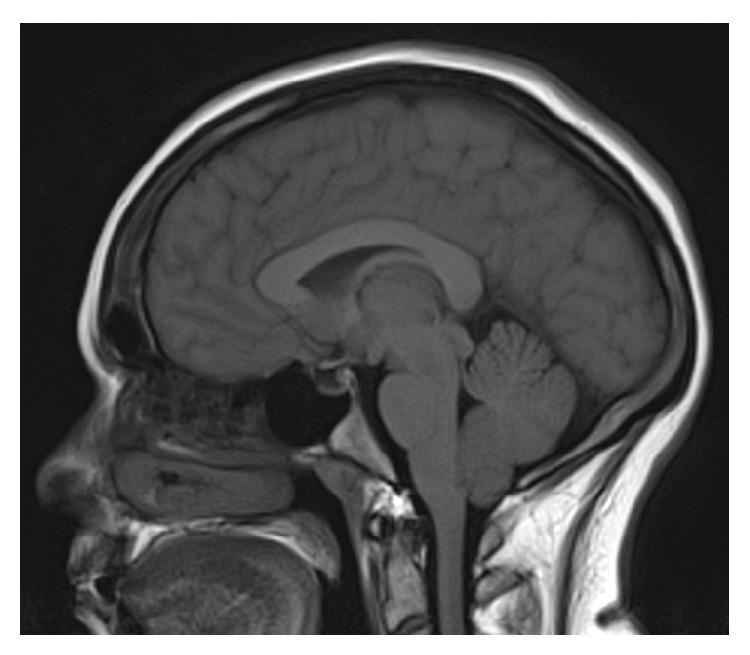
Sagittal MRI at diagnosis demonstrated 4 mm of cerebellar tonsillar descent.

**Figure 2:**
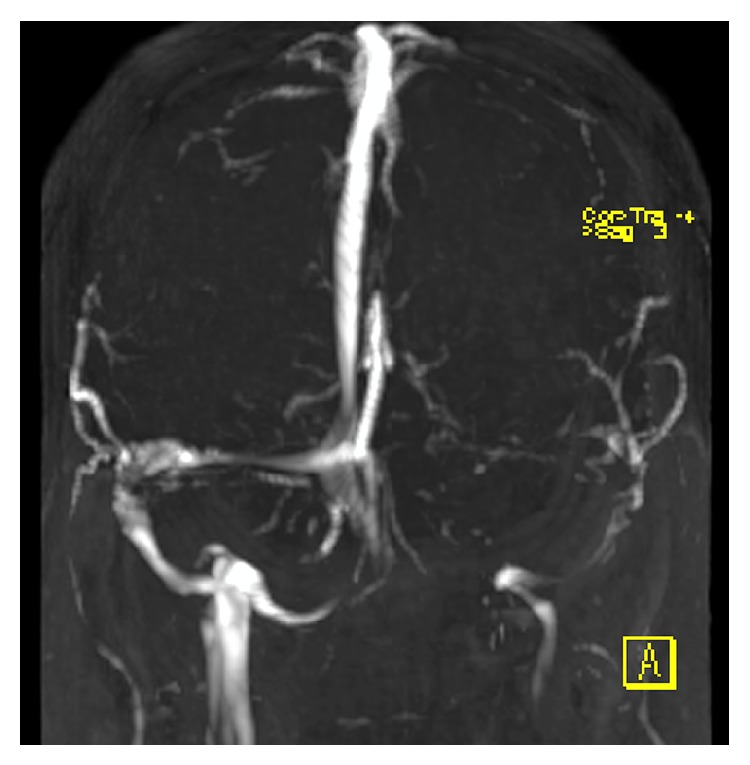
Time of flight MR venogram (anterior view) demonstrating aplastic L transverse sinus with R transverse sinus stenosis.

A 30-year-old female with a BMI of 37.9 was diagnosed with IIH seven months prior to this presentation. Her MRI at diagnosis demonstrated 4 mm of cerebellar tonsillar descent (Figure 1) with flattening of the posterior globes, mild distension of the optic nerve sheaths, and a partially empty sella turcica. At the time of diagnosis, her LP revealed an opening pressure of 42 cm H_2_O. She was prescribed regular oral acetazolamide and regularly underwent therapeutic drainage of 15 mL of cerebrospinal fluid (CSF) for symptom control without complication. Her opening pressures were consistently between 35 and 50 cm H_2_O. MR venogram demonstrated an aplastic left transverse sinus with a right transverse sinus stenosis with extrinsic compression, for which she was being considered for a stenting procedure (Figure 2).

She presented to the emergency department with four days of severe headache, nausea, and vomiting. Her past history included renal tubular acidosis diagnosed two years prior and treated with sodium bicarbonate and electrolyte replacement. In the emergency department she had bilateral papilloedema with no new focal neurology. She was normothermic with a CRP of 2.8 mg/L. A therapeutic LP was performed with an opening pressure of 43 cm H_2_O. 18 mL of clear CSF was drained and she was admitted to the neurology ward for observation and analgesia.

Overnight she had a medical emergency team (MET) call for hypotension attributed to hypovolemia due to ongoing vomiting, which was treated with intravenous fluids. She had a further MET call overnight for transient visual disturbance and reported being unable to appreciate light/dark and had severe headache. Her pupils were bilaterally dilated but responsive to light. She was alert and interactive and had no motor deficits. The visual changes resolved without intervention and she was taken for a CT venogram, which demonstrated bilateral tentorial herniation with 10 mm of cerebellar tonsillar herniation. There were no features of dural venous thrombosis.

**Figure 3:**
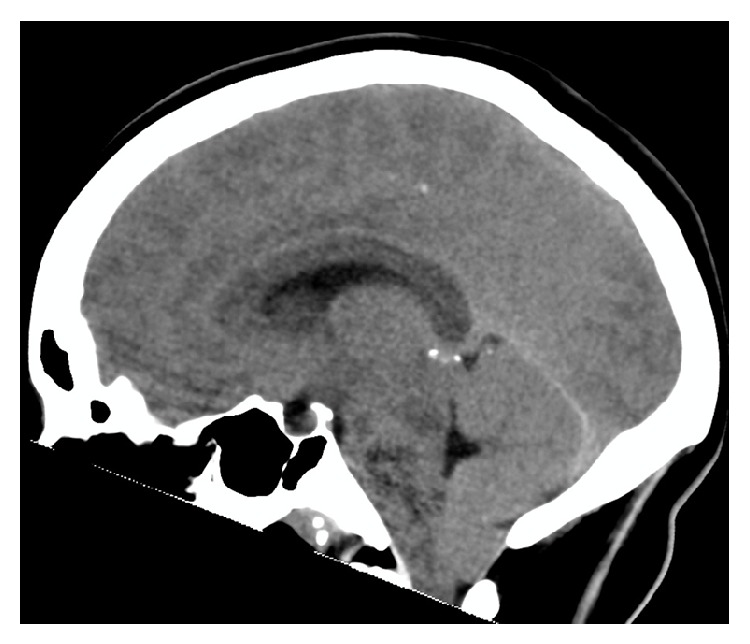
Sagittal CT scan demonstrating 10 mm of tonsillar descent with posterior fossa crowding.

She was reviewed on the morning of day 1 and found to be agitated, Glasgow Coma Scale (GCS) 13 (M6, V4, E3), with a fixed dilated right pupil 7 mm. Her left pupil was 5 mm and reactive. Plantar response was upgoing bilaterally and her neck was hyperextended with extreme pain on neck flexion. Heart rate was 65 bpm and blood pressure was 112/66 mmHg. She was transferred urgently to the intensive care unit (ICU) and intubated for anticipated decline whilst awaiting a CT brain. During the CT scan she became bradycardic with a heart rate of 30 and asystolic pauses, treated with osmotic therapy of 10 mL 20% sodium chloride. Her BP was then recorded as 147/77 mmHg. The CT scan again showed 10 mm of tonsillar descent and an increase in fourth ventricle size with posterior fossa crowding (Figure 3). Her initial LP result showed a mildly elevated white cell count of 7 × 10^6^/L (normal < 5 × 10^6^/L) and she was commenced on aciclovir empirically.

She was treated with a further 20 mL of 20% sodium chloride and taken for urgent insertion of an external ventricular drain (EVD), a technically difficult procedure due to small ventricular size, which rapidly improved right pupil reactivity although it remained sluggish. She returned to ICU with the EVD on free drainage 15 cm above the tragus. Her ICP remained persistently high at >45 cm H_2_O and the EVD was lowered to 10 cm to allow further drainage. As her pressure on previous LPs was 35–50 cm H_2_O, a target of 35 cm H_2_O was agreed upon for initial management.

On day 2 she had persistently high ICP >60 cm H_2_O despite aggressive management with hyperventilation to PaCO_2_ of 35 mmHg, osmotic therapy with 20% sodium chloride, deep sedation with morphine and midazolam, and aggressive temperature management, targeting normothermia. Her ICP did not respond to intermittent paralysis; thus a paralytic infusion was not commenced. CT scan demonstrated 12 mm of tonsillar herniation. She was treated with 16 mg dexamethasone and taken for rescue bifrontal decompressive craniectomy. The brain was noted to be grossly swollen, hyperaemic, and herniating with marked cortical venous distension. The craniectomy was insufficient alone to provide cerebral decompression due to the inelasticity of the dura; thus the latter was opened with multiple slits during which a cortical vein was lacerated requiring diathermy. The EVD was removed and a fibre-optic pressure monitor was placed.

Following this, her ICP remained <10 cm H_2_O. Postoperative CT scan showed 8 mm of tonsillar herniation and a significant increase in size of the third ventricle. CSF culture was negative and viral PCR for HSV1, HSV2, enterovirus, and VZV were also negative and her empiric aciclovir was ceased.

On day 3 high dose sedation was ceased with slow recovery including transient diabetes insipidus treated with desmopressin and reintubation for poor sputum clearance. She had a gradual neurological improvement and was discharged to the neurology ward with a GCS 15 and no focal neurology on day 15 of her admission.

Unfortunately she developed CSF rhinorrhoea and pneumocephalus due to a CSF fistula through the frontal air sinus requiring surgical repair. She subsequently developed a venous infarct complicated by seizures and the requirement for intubation for reduced level of consciousness.

**Figure 4:**
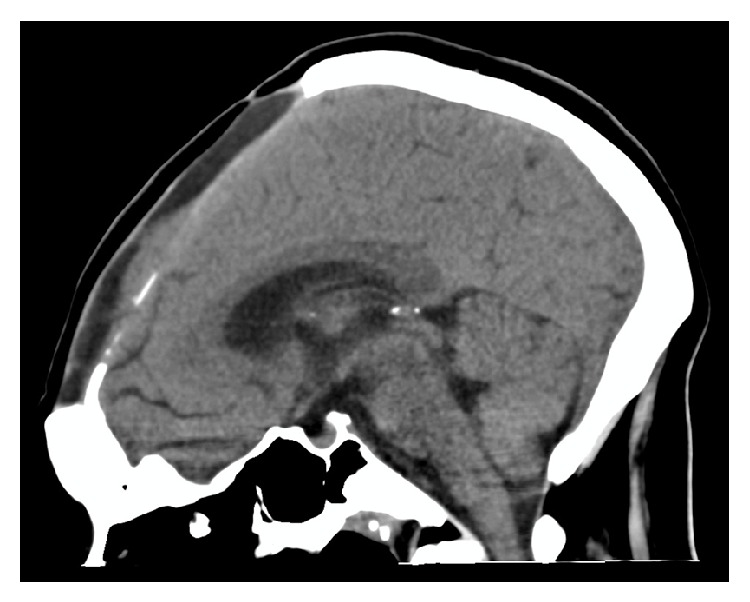
Sagittal CT demonstrating normal cerebellar tonsillar position after decompressive craniectomy.

After rehabilitation, she was discharged home on day 69 after admission and was able to function independently, although she had not returned to work. At the time of publication she is undergoing neuropsychological testing to evaluate her readiness to return to work. Her cerebellar tonsils had returned to a normal position on a follow-up outpatient CT scan (Figure 4).

## 3. Discussion

Given the rarity of cerebellar tonsillar herniation in this setting, literature regarding management was not available. Management was thus based upon physiological principles such as the Monro-Kellie hypothesis and consensus between medical teams.

There were several unusual aspects to her presentation. Firstly, she had a known vascular malformation with an aplastic left transverse sinus and stenosis of the right transverse sinus. Vascular abnormalities do not preclude the diagnosis of IIH and have been implicated in the pathogenesis [6]. Resolution of symptoms has been reported in those with dural venous sinus stenting [7], the procedure she was being considered for. She also already had 4 mm of cerebellar tonsillar descent documented on an MRI six months prior to presentation; however criterion for identifying a clearly pathological Chiari 1 malformation is 5 mm [8]. It has previously been suggested that the combination of low lying tonsils and raised ICP potentially contributed to herniation in this setting [5]. Despite mild elevation of her CSF white cell count and empiric treatment with aciclovir, her viral PCR returned negative and her clinical presentation was not consistent with encephalitis.

Prior to her urgent transfer to ICU, there was a high index of suspicion of raised ICP given her fixed dilated pupil. However, at the time she was conscious, moving all limbs, and interactive despite severe pain. This was considered unusual compared with the significantly decreased level of consciousness associated with raised ICP in traumatic brain injury (TBI). It is therefore pertinent to recognize that TBI represents a syndrome comprising both the primary brain injury and secondary rise in ICP with hematoma formation and/or oedema.

There were several differences in this patient compared with the two previously reported cases. Firstly, herniation in IIH usually occurs as a late complication following lumbar peritoneal shunting [9]. This is consistent with the hypothesis that a reduction in pressure gradually restores normal brain compliance [4]. Secondly, in the rare case of herniation following LP the time course has previously been reported as occurring within minutes [5], whereas this case occurred over a period of 14 hours. This may suggest an alternative cause of the rise in ICP, perhaps a rebound phenomenon due to her inability to tolerate oral acetazolamide due to vomiting. Acetazolamide has been demonstrated to significantly reduce intracranial pressure [10].

In the absence of supportive literature, management followed local practice of TBI patients with raised ICP. Medical management initially involved sedation, analgesia, PaCO_2_ control, targeted temperature management, and intermittent osmotic therapy with 20% sodium chloride. As the above interventions were not able to control her ICP, barbiturate coma was unlikely to be successful, and a surgical option was pursued.

Issues arose however regarding the ICP target once the EVD was inserted given the fact that her usual pressures were between 35 and 50 cm H_2_O. An initial target of 35 cm H_2_O was decided upon to provide a moderate reduction in pressure, without requiring excessive vasopressor doses to achieve an adequate cerebral perfusion pressure. The goal was to gradually reduce this down to 30 cm H_2_O.

The surgical procedure to control her critical intracranial hypertension was performed to provide rapid and life-saving control. It appeared that the aplastic left transverse sinus and consequent anomalous posterior cranial fossa drainage led to grossly hypertrophied cerebellar and circular venous sinuses. Foramen magnum decompression and cerebellar tonsillar disimpaction were considered in addition to bifrontal craniectomy. However, these were not performed acutely because her aberrant venous anatomy presented an unacceptably high risk of uncontrollable venous sinus haemorrhage.

**Figure 5:**
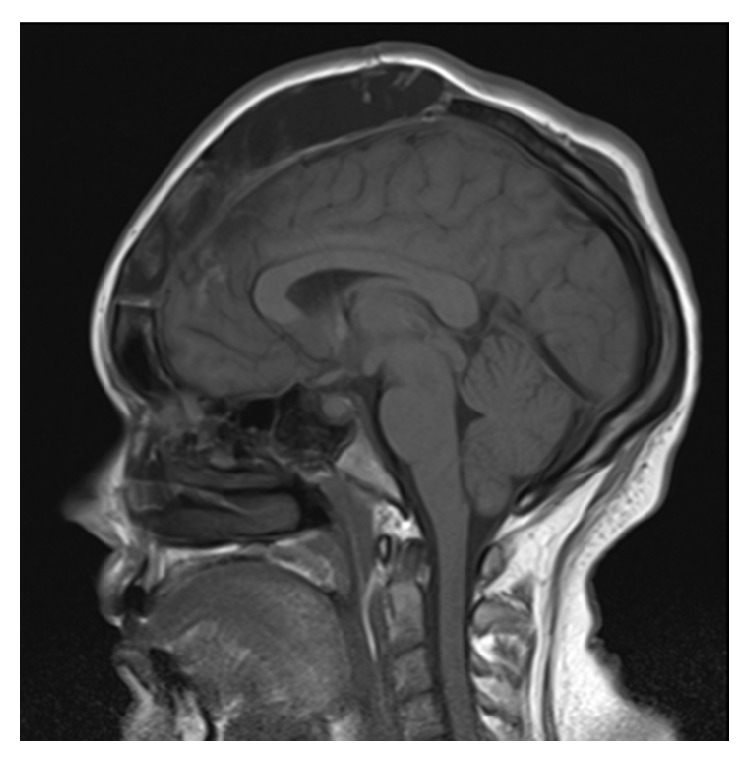
Sagittal MRI 3 weeks after decompressive craniectomy.

Decompressive craniectomy was performed as a rescue procedure. In a recent trial, worse functional outcomes have been identified in TBI patients with second line decompressive craniectomy [11]. It has been postulated that this is the result of neuronal injury due to axonal stretch with herniation via the craniectomy site. Given the hypothesis regarding reduced brain compliance in IIH [4] it is possible that this is protective against further neuronal injury due to stretch. In this case, the decompressive craniectomy not only brought her intractable intracranial hypertension under immediate control but also resulted in disimpaction of her cerebellar tonsillar herniation (Figure 5).

## 4. Conclusion

In this patient with IIH who had cerebellar tonsillar herniation following LP, management similar to traumatic intracranial hypertension was successful. ICP targets in this setting are unknown; however we recommend a moderate reduction compared with baseline pressures as long as focal neurology resolves. Given the rapid improvement in ICP control and favourable neurological outcome, we recommend that decompressive craniectomy be considered, especially given the possible protection against further brain injury from axonal stretch due to reduced brain compliance.
